# Administration of Enalapril Started Late in Life Attenuates Hypertrophy and Oxidative Stress Burden, Increases Mitochondrial Mass, and Modulates Mitochondrial Quality Control Signaling in the Rat Heart

**DOI:** 10.3390/biom8040177

**Published:** 2018-12-17

**Authors:** Anna Picca, Giuseppe Sirago, Vito Pesce, Angela Maria Serena Lezza, Riccardo Calvani, Maurizio Bossola, Emanuele Rocco Villani, Francesco Landi, Christiaan Leeuwenburgh, Roberto Bernabei, Christy S. Carter, Emanuele Marzetti

**Affiliations:** 1 Fondazione Policlinico Universitario “Agostino Gemelli” IRCSS, Università Cattolica del Sacro Cuore, L.go A. Gemelli 1, 00168 Rome, Italy; anna.picca1@gmail.com (A.P.); mauriziobossola@gmail.com (M.B.); francesco.landi@unicatt.it (F.L.); roberto.bernabei@unicatt.it (R.B.); emanuele.marzetti@policlinicogemelli.it (E.M.); 2 Institute of Internal Medicine and Geriatrics, Università Cattolica del Sacro Cuore, 00168 Rome, Italy; emanuele.rocco.villani@gmail.com; 3 Department of Biosciences, Biotechnologies and Biopharmaceutics, University of Bari, 70125 Bari, Italy; giuseppesirago88@gmail.com (G.S.); vito.pesce@uniba.it (V.P.); angelamariaserena.lezza@uniba.it (A.M.S.L.); 4 Institute of Surgical Sciences, Fondazione Policlinico Universitario “Agostino Gemelli” IRCSS, 00168 Rome, Italy; 5 Department of Aging and Geriatric Research, Institute on Aging, Division of Biology of Aging, University of Florida, Gainesville, FL 32611, USA; cleeuwen@ufl.edu; 6 Department of Medicine, Division of Gerontology, Geriatrics and Palliative Care, Nathan Shock Center of Excellence in the Basic Biology of Aging, University of Alabama at Birmingham, Birmingham, AL 35205, USA; cartercs@uabmc.edu

**Keywords:** mitochondrial quality control, mitochondrial biogenesis, mitochondrial dynamics, mitophagy, mtDNA, TFAM binding, oxidative lesions, mtDNA damage, cardioprotection, peroxiredoxin

## Abstract

Mitochondrial dysfunction is a relevant mechanism in cardiac aging. Here, we investigated the effects of late-life enalapril administration at a non-antihypertensive dose on mitochondrial genomic stability, oxidative damage, and mitochondrial quality control (MQC) signaling in the hearts of aged rats. The protein expression of selected mediators (i.e., mitochondrial antioxidant enzymes, energy metabolism, mitochondrial biogenesis, dynamics, and autophagy) was measured in old rats randomly assigned to receive enalapril (*n* = 8) or placebo (*n* = 8) from 24 to 27 months of age. We also assessed mitochondrial DNA (mtDNA) content, citrate synthase activity, oxidative lesions to protein and mtDNA (i.e., carbonyls and the abundance of mtDNA^4834^ deletion), and the mitochondrial transcription factor A (TFAM) binding to specific mtDNA regions. Enalapril attenuated cardiac hypertrophy and oxidative stress-derived damage (mtDNA oxidation, mtDNA^4834^ deletion, and protein carbonylation), while increasing mitochondrial antioxidant defenses. The binding of mitochondrial transcription factor A to mtDNA regions involved in replication and deletion generation was enhanced following enalapril administration. Increased mitochondrial mass as well as mitochondriogenesis and autophagy signaling were found in enalapril-treated rats. Late-life enalapril administration mitigates age-dependent cardiac hypertrophy and oxidative damage, while increasing mitochondrial mass and modulating MQC signaling. Further analyses are needed to conclusively establish whether enalapril may offer cardioprotection during aging.

## 1. Introduction

The intimate mechanisms that underlie cardiac aging are yet to be fully deciphered. Yet, wide consensus exists on the central role played by mitochondrial dysfunction [1]. Besides their function in cellular energy provision, these organelles also serve as the hub for many other activities, including metabolic signaling, regulation of programmed cell death, calcium and iron buffering, and iron-sulfur cluster and heme biosynthesis [2]. As such, the maintenance of well-performing mitochondria is instrumental to cell viability. Like neurons and skeletal myocytes, cardiomyocytes are post-mitotic cells with very limited regenerative capacity and are thus devoid of a replicative dilution of damage [3]. Therefore, repair and recycling processes are essential for mitochondrial quality control (MQC), which is accomplished through an integrated network of pathways operating sequentially from individual molecules to the whole organelle [4].

During aging, the generation of reactive oxygen species (ROS) increases in cardiomyocyte mitochondria [5]. The concomitant decrease in the efficiency of antioxidant defenses contributes to establishing an oxidant environment [5,6], which is thought to play a key role in mitochondrial dysfunction during cardiac aging [7]. Indeed, mitochondrial macromolecules, including mitochondrial DNA (mtDNA), are immediate targets of ROS. As a result of persistent oxidative stress, both quantitative and qualitative mtDNA alterations may occur [8], which impact mtDNA structure and function. Noticeably, the frequency of the common 4977-bp mtDNA deletion, a typical oxidative stress-derived lesion [9], increases with age in the human heart and is 5- to 15-fold higher in people over 40 years of age compared with younger adults [10,11].

Oxidation of DNA bases is another extensively investigated mutation associated with aging [12]. Of the four DNA bases, guanine has the lowest reduction potential and is the most susceptible to oxidation [13]. Oxidized guanine accumulates in the aging heart, especially in the GC-rich mtDNA [14], and can induce transversion mutations [15]. Furthermore, a direct relationship between base oxidation and the generation of mtDNA deletions has been proposed [16], such that oxidized bases trigger the recombination of mtDNA segments in the presence of direct repeats flanking the deletion regions by facilitating DNA strand separation [16].

The mitochondrial transcription factor A (TFAM), a nuclear-encoded histone-like protein that was originally attributed a role in mtDNA replication and transcription [17], may also be involved in the sensing and repair of mtDNA oxidative damage [18]. The mechanism whereby TFAM intervenes in mtDNA repair is presently unknown. However, TFAM seems to preferentially bind to mtDNA damage hot spots [19]. This finding leads to the hypothesis that TFAM binding might hinder mtDNA repair by limiting the access of repairing enzymes to the site of lesions and/or by trapping the accessed enzymes and preventing their activities [19].

Previous findings from our group support a mitochondrial protective effect of angiotensin-converting enzyme (ACE) inhibitors in the skeletal muscle of old rats [20,21]. Furthermore, Piotrkowski et al. [22] showed that enalapril given at a non-antihypertensive dose prevented cardiac hypertrophy and ameliorated cardiomyocyte mitochondrial dysfunction in spontaneously hypertensive rats. Whether ACE inhibition affects oxidative stress, mtDNA homeostasis, and MQC in the aging heart and thus offers cardioprotection during aging is currently unknown.

The present study was therefore undertaken to investigate the effects of enalapril administration started late in life on mitochondrial genomic stability, oxidative damage, and MQC signaling in the rat heart. In particular, we tested the hypothesis that enalapril administration at a non-antihypertensive dose would attenuate oxidative stress and some of its consequences (i.e., mtDNA damage, mtDNA^4834^ deletion, and protein carbonylation), increase mitochondrial mass, promote mitochondrial fusion, and stimulate autophagy.

## 2. Materials and Methods

### 2.1. Animals

Fischer 344×Brown Norway F1 hybrid rats were purchased from the National Institute on Aging Colony at Harlan Industries (Indianapolis, IN, USA). Rats were received at 22 months of age and housed individually on a 12-h light/dark cycle in a specific pathogen-free facility accredited by the American Association for Accreditation of Laboratory Animal Care. Health status, body weight (BW), and food intake were monitored daily. Rats were randomly assigned to receive 20 mg kg^−1^ day^−1^ enalapril (*n* = 8) or placebo (*n* = 8) from 24 to 27 months of age. The enalapril dose did not modify blood pressure according to pilot experiments conducted in our laboratory [23]. Drug delivery was accomplished by compounding treatments into bacon-flavored food tablets (Bio-Serv, Frenchtown, NJ, USA). Placebo-containing food tablets were identical to those delivering enalapril, except for drug omission. Drug- and placebo-containing tablets were administered separately from the standard chow. Drug doses were tailored daily according to the animal’s weight. All rats consumed the whole treatment tablet at each meal. Animals were anesthetized before being sacrificed, weighed, and the heart was immediately removed, weighed, snap-frozen in isopentane cooled by liquid nitrogen, and stored at −80 °C until analysis. The study and experimental protocols were approved by the University of Florida’s Institutional Animal Care and Use Committee (protocol number 200801599) and complied with the International Guiding Principles for Biomedical Research Involving Animals.

### 2.2. Western Immunoblotting

Western blot experiments were carried out on heart homogenates from each animal of the two experimental groups. Ten μg of total proteins were separated by sodium dodecyl sulfate polyacrylamide gel electrophoresis (SDS-PAGE) and subsequently electroblotted onto polyvinylidenefluoride (PVDF) hybond-P membranes (GE Healthcare, Buckinghamshire, UK), using the Criterion Blotter (Bio-Rad Laboratories, Hercules, CA, USA). After the protein transfer, membranes were probed overnight at 4 °C with primary antibodies targeting the following proteins: protein kinase B (AKT), phospho-AKT (P-AKT), 5′ AMP-activated protein kinase (AMPK), phospho-AMPK (P-AMPK), Beclin 1, dynamin-related protein 1 (Drp1), Forkhead boX O3a (FoXO3a), phospho-FoXO (P-FoXO3a), mitofusin 2 (Mfn2), manganese superoxide dismutase (MnSOD), peroxisome proliferator-activated receptor gamma coactivator 1-alpha (PGC-1α), peroxiredoxin III (PrxIII), oxidized PrxIII (Prx-SO_3_), sequestosome 1 (SQSTM1) p62 protein, TFAM, and glyceraldehyde 3-phosphate dehydrogenase (GAPDH, loading control). Technical specifications of the primary antibodies used are listed in Table 1. The following day, membranes were incubated for 1 h at room temperature with appropriate peroxidase-conjugated secondary antibodies (Santa Cruz Biotechnology, Santa Cruz, CA). Blots were visualized using the ECL Plus Western Blotting Detection Reagents and ECL films (GE Healthcare). Autoradiographs were acquired by the ChemiDoc MP Imaging System and analyzed by Quantity One software (Bio-Rad Laboratories). The densitometric value of optical density (OD) units of each protein band immunodetected was then related to the corresponding GAPDH signal intensity (loading control) and normalized by comparison with the placebo group.

### 2.3. Quantification of Mitochondrial DNA Content

Genomic DNA was isolated from the heart of each animal from the two experimental groups and checked for quality as reported previously [24]. Thirty-40 mg of heart tissue were used. Total DNA was quantified using a NanoDrop 2000/2000c spectrophotometer (Thermo Fisher Scientific, Waltham, MA, USA) and integrity was verified by gel electrophoresis on 0.8% agarose gel in 1× TBE (90 mM Tris-borate pH 7.4, 90 mM boric acid, 2.5 mM EDTA). Mitochondrial DNA content was measured using a quantitative real-time polymerase chain reaction (qRT-PCR), as described elsewhere [25]. Quantitative RT-PCR amplification reactions were carried out on an ABI PRISM 7300 Sequence Detection System (Applied Biosystems, Foster City, CA, USA) using SYBR Green chemistry (Power SYBR Green Master Mix, Thermo Fisher Scientific). Primers for the rat mitochondrial D-loop region and for the rat nuclear β-actin gene were designed with the Primer Express 3.0 software (Applied Biosystems) (Table 2). The method was validated by primer-limiting experiments (200 nM for each primer pair concentrations) and by evaluating the equal reaction efficiency of the two amplicons. A melting curve analysis and gel electrophoresis were used to control the amplification specificity. Each sample was analyzed in triplicate in 25 µL final volume. The reaction mixture consisted of iTaq SYBR Green Supermix PCR 1× Master Mix (Bio-Rad Laboratories), 0.2 µM forward and reverse primers, and DNA template (25 ng). The amplification proceeded for 40 cycles. The quantification of the relative mtDNA content was performed according to the Pfaffl mathematical model [26]. In particular, the difference in threshold cycle values (ΔCt, namely Ct D-loop - Ct β-actin) was used as a measure of the relative abundance of the mitochondrial genome. To compare mtDNA quantity between experimental groups, the relative amount of mtDNA to nuclear DNA was calculated using the following equation: *R* = 2^ΔΔCt^, where *R* is the calculated ratio and ΔΔCt is the ΔCt analyzed class-ΔCt reference class value, with the placebo group taken as the reference.

### 2.4. Relative Quantification of Mitochondrial DNA^4834^ Deletion

The level of the mtDNA^4834^ deletion was measured by qRT-PCR via SYBR Green chemistry on an ABI PRISM 7300 Sequence Detection System (Applied Biosystems) as previously reported [25]. Primers were designed with the Primer Express 3.0 software (Applied Biosystems) for the rat mtDNA 4.8-kb deleted region and for the rat mtDNA D-loop region, generally undeleted (Table 2).

The method was validated by primer-limiting experiments and by evaluating the equal reaction efficiency of the two amplicons. Amplification specificity was controlled by a melting curve analysis and following gel electrophoresis. Each sample was analyzed in triplicate in 25 µL final volume and fluorescence spectra were monitored. The reaction mixture consisted of iTaq SYBR Green Supermix PCR 1× Master Mix (Bio-Rad Laboratories), 0.2 µM forward and reverse primers, and DNA template (2.5 µL of diluted 1:10). After 10 min of denaturation at 95 °C, amplification proceeded for 40 cycles, each consisting of denaturation at 95 °C for 15 s, annealing, and extension at 60 °C for 1 min. The relative abundance of the 4.8-kb deleted mtDNA in placebo and enalapril-treated rats, all normalized to the corresponding total mtDNA, was calculated according to the Pfaffl mathematical model [26] using the equation *R* = 2^ΔΔCt^, as described above.

### 2.5. Measurement of Citrate Synthase Activity

Total proteins were purified from 15 mg of frozen heart samples by homogenization in a buffer containing 100 mM mannitol, 1 mM ATP, 0.2% bovine serum albumin (BSA), 100 mM KCl, 3 mM MgCl_2_, 5 mM Tris-buffer, 1 mM EDTA, pH 7.4. Protein concentration was determined by the Bradford method [27] according to the supplier’s instructions (Bio-Rad Laboratories). Citrate synthase (CS) activity (µmol × min^−1^ × g tissue^−1^) was determined in tissue homogenates according to the method developed by Srere [28]. Briefly, 100 µg of total proteins were incubated in 1 mL of assay buffer containing 0.31 mM acetyl-CoA, 100 mM Tris buffer (pH 8.1), 0.25% Triton X-100, 0.1 mM 5,5′-dithio-bis-2-nitrobenzoic acid, and 0.5 mM oxaloacetate at 30 °C. Citrate synthase activity was determined spectrophotometrically by measuring the rate of production of thionitrobenzoic acid at 412 nm.

### 2.6. Detection of Mitochondrial DNA Oxidative Damage

To detect oxidative base modifications in mtDNA, we adapted the PCR assay developed by Pastukh et al. [29], as previously described [19]. Analyses focused on short sequences of specific mtDNA genes: A portion of the cytochrome b gene (complex III of the electron transport chain); the OriL origin together with a portion of the *CO I* gene; a portion of the D-loop with the OriH origin of replication; and a sequence including the direct repeat 1 of the 4.8-kb deletion. Primers used to amplify the sequences of interest are listed in Table 2. The basis of the assay is the treatment of DNA with the enzyme formamidopyrimidine [fapy]-DNA glycosylase (Fpg) (New England Biolabs, Beverly, MA, USA) which results in strand cleavage at sites of oxidized purines, thereby creating single-strand breaks that block PCR amplification. Differences in PCR amplification between Fpg-treated and untreated DNA are thus a specific indicator of the presence of oxidative base damage. The Fpg cleavage reaction was performed by incubating 250 ng of DNA with 8 U of Fpg in 1× NEBuffer 1 (10 mM Bis/Tris propane-HCl, 10 mM MgCl_2_, 1 mM DTT, pH 7.0) and 100 μg/mL BSA in a volume of 50 μL. Incubations were carried out at 37 °C for 1 h. [fapy]-DNA glycosylase was then inactivated by heating at 60 °C for 5 min. An aliquot of 10 ng of DNA was used for the PCR assay to detect Fpg-sensitive cleavage sites. Data are presented as the fraction of intact DNA, calculated as the ratio of band intensities of Fpg-treated and untreated samples [30].

### 2.7. Analysis of TFAM Binding to mtDNA by Mitochondrial Immunoprecipitation

The binding of TFAM to specific regions of mtDNA was analyzed using mtDNA immunoprecipitation (mIP). The measurement of the relative amounts of mtDNA immunoprecipitated by TFAM was carried out by qRT-PCR as previously described [25]. Three primer pairs, listed in Table 2, were designed to include, respectively, a part of the D-loop with the OriH origin of replication; the Ori-L origin together with a portion of the *CO I* gene; and a part of the ATPase 6 gene containing the direct repeat 1 of the 4.8-kb deletion. Input and mIP mtDNAs were subjected to qRT-PCR amplification reactions via SYBR Green chemistry on an ABI PRISM 7000 Sequence Detection System (Applied Biosystems). The reaction mixture consisted of iTaq SYBR Green Supermix PCR 1× Master Mix (Bio-Rad Laboratories), 0.2 μM forward and reverse primers, and 2.5 μL of the input or immunoprecipitated DNA aliquots with or without anti-TFAM antibodies (diluted 1:10). After a 10-min denaturation at 95 °C, samples were amplified for 40 cycles, each consisting of denaturation at 95 °C for 15 s, annealing, and extension at 60 °C for 1 min. The calculation of the amount of TFAM-bound mtDNA was performed according to the formula 2^ΔCTx^-2^ΔCTb^, where ΔCTx is the difference between the CT values of the input and the immunoprecipitated sample and ΔCTb is the CT difference between the CT values of the input and the no-antibody sample [31].

### 2.8. Determination of Protein Carbonylation

The levels of protein-bound carbonyls were evaluated by means of the OxyBlot Protein Oxidation Detection Kit (Millipore, Billerica, MA, USA) according to the manufacturer’s instructions. Equal amounts of total proteins (10 μg) were treated with 6% SDS in 10 μL final volume. Carbonyl groups in the protein side chains were derivatized with 2,4-dinitrophenyl hydrazine (DNPH) to form 2,4-dinitrophenyl hydrazone (DNP hydrazone) following an incubation of 15 min at room temperature. Negative controls were obtained by incubating protein extracts with a derivatization control solution at the same incubating conditions. After blocking the derivatization reaction with a neutralization solution, β-mercaptoethanol was added at a final concentration of 0.74 M. Protein samples were separated on a 4 to 15% Criterion TGX Stain-Free Precast Gels (Bio-Rad Laboratories) and processed by Western immunoblotting. Blotted membranes were blocked in 1% BSA in PBS 1×, 0.05% Tween 20 and incubated overnight at 4 °C with a specific antibody against DNP hydrazone (1:300 in 1% BSA in 1× PBS, 0.05% Tween 20) and for 1 h at room temperature with the secondary antibody (1:150 in 1% BSA in 1× PBS, 0.05% Tween 20). The normalization of protein samples was performed with the fluorescent detection in Stain Free Blot using a ChemiDoc MP imaging system (Bio-Rad Laboratories) on the PVDF membrane. The quantification of carbonylated proteins was performed on Image Lab 6.0 software (Bio-Rad Laboratories) using the “Total Lane Protein” setting.

### 2.9. Statistical Analysis

The normal distribution of data was ascertained through the Kolmogorov–Smirnov test. Subsequently, comparisons between experimental groups (i.e., placebo- and enalapril-treated rats) were performed by *t*-test statistics. Changes in food intake over time in the two treatment groups were analyzed by two-way analysis of variance for repeated measures. All analyses were performed using the GraphPrism 5.03 software (GraphPad Software, Inc., San Diego, CA, USA), with the statistical significance set at *p* < 0.05.

## 3. Results

### 3.1. Morphological and Physiological Characteristics of Experimental Animals

Body weight was comparable between treatment groups both at baseline and at the time of sacrifice (Table 3). In contrast, the heart weight, either absolute or adjusted by BW, was significantly lower in enalapril-treated rats compared with controls (*p* = 0.0359 for both). Food consumption was slightly lower in rats receiving enalapril and declined to a similar extent in both groups over time.

### 3.2. Oxidative Stress and Mitochondrial DNA Lesions

One relevant outcome of mitochondrial dysfunction is oxidative stress. Therefore, we estimated the oxidative burden of our samples by determining the protein expression of two mitochondrial ROS-scavenging enzymes and the level of protein-bound carbonyls in the heart of the two rat groups.

**Figure 1:**
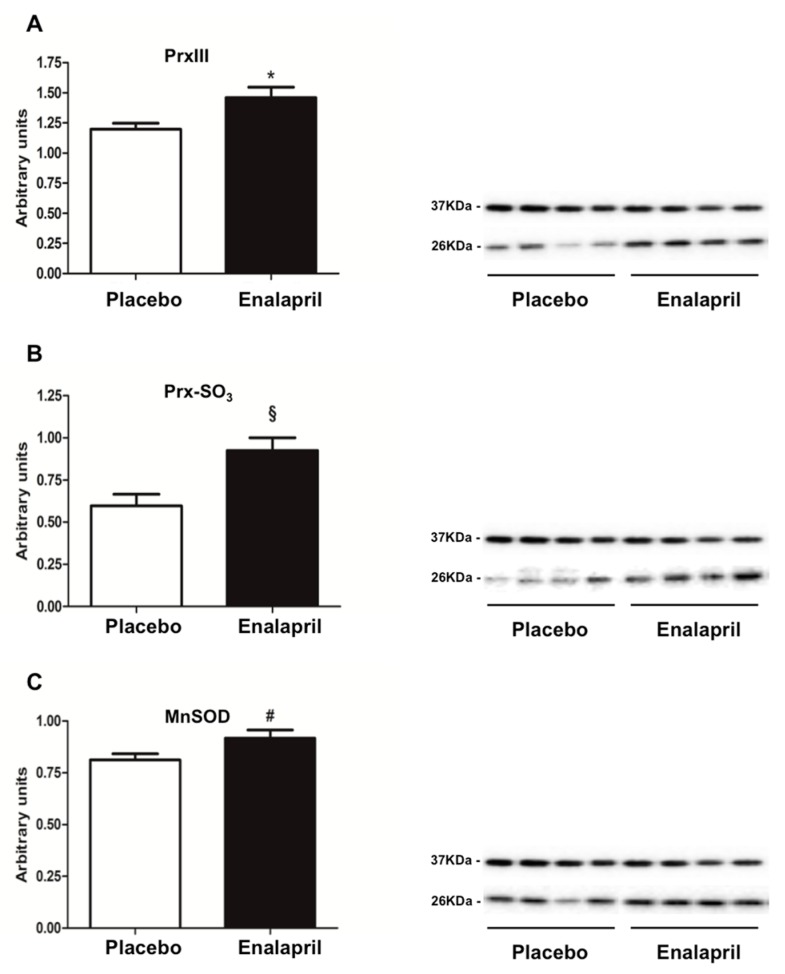
Protein expression of the mitochondrial antioxidant enzymes (**A**) peroxiredoxin III (Prx-III), (**B**) its oxidized form Prx-SO_3_, and (**C**) manganese superoxide dismutase (MnSOD) in heart samples of old rats treated with placebo (*n* = 8) or enalapril (*n* = 8). Bars represent mean values (± standard error of the mean) in the two experimental groups. Values are expressed in arbitrary units. Representative blots are shown. * *p* = 0.0140 vs. placebo; ^§^ *p* = 0.0029 vs. placebo; ^#^ *p* = 0.0450 vs. placebo.

**Figure 2:**
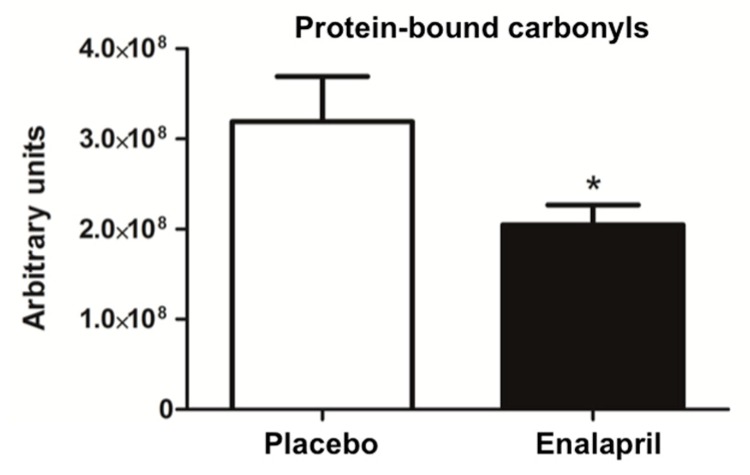
Levels of protein-bound carbonyls in heart samples of old rats treated with placebo (*n* = 8) or enalapril (*n* = 8). Bars represent mean values (± standard error of the mean) in the two experimental groups. Values are expressed in arbitrary units. * *p* = 0.0457 vs. placebo.

The content of mitochondrial PrxIII was increased in rats treated with enalapril (*p* = 0.0140; Figure 1A). Despite an overall reduced level of protein-bound carbonyls in the heart of enalapril-treated rats (*p* = 0.0457; Figure 2), an accumulation of the oxidized sulphonic form of Prx III (Prx-SO_3_) was identified in the same experimental group (*p* = 0.0029; Figure 1B). In contrast, the expression of MnSOD was higher in enalapril-treated rats compared with the placebo group (*p* = 0.0450; Figure 1C).

**Figure 3:**
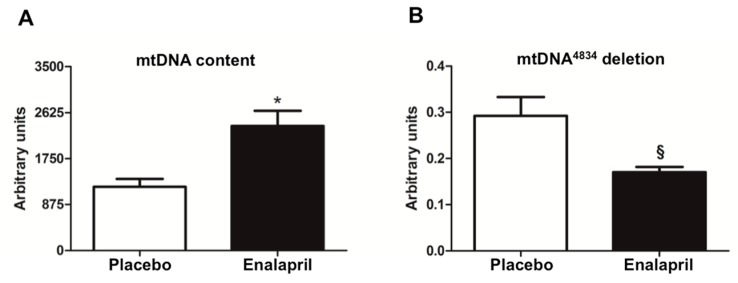
Determination of (**A**) mtDNA content and (**B**) mtDNA^4834^ deletion abundance in heart samples of old rats treated with placebo (*n* = 8) or enalapril (*n* = 8). Bars represent mean values (± standard error of the mean) in the two experimental groups. Values are expressed in arbitrary units. * *p* = 0.001 vs. placebo; ^§^ *p* = 0.0173 vs. placebo.

Since increased mitochondrial biogenesis does not always translate into a gain of function [32], we determined the content of mtDNA and quantified the abundance of mtDNA^4834^ deletion and mtDNA oxidative damage in the two rat groups. Greater mtDNA content was detected in enalapril-treated rats compared with controls (*p* = 0.001; Figure 3A), together with decreased levels of deleted molecules in the same rat group (*p* = 0.0173; Figure 3B), suggesting that enalapril administration might protect against mtDNA damage through increased mtDNA replication.

**Figure 4:**
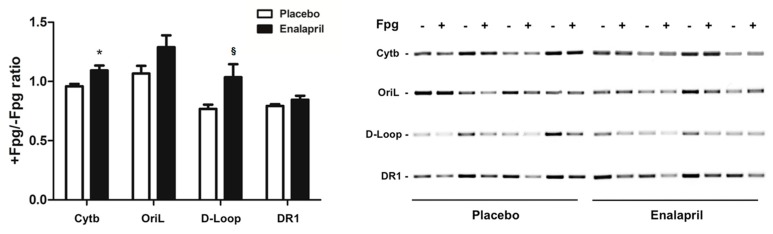
Analysis of oxidative base damage to specific regions of mtDNA in heart samples of old rats treated with placebo (*n* = 8) or enalapril (*n* = 8). Bars represent mean values (± standard error of the mean) in the two experimental groups. Data are presented as the ratio between Fpg-treated and non-treated samples and show the fraction of mtDNA free of oxidative damage. Representative blots are shown. * *p* = 0.0146 vs. placebo; ^§^ *p* = 0.0354 vs. placebo.

The determination of mtDNA oxidative damage provided insightful observations. We focused our analysis on short mtDNA sequences including functional regions that regulate mtDNA replication and the generation of the mtDNA^4834^ deletion. The Cytb and D-Loop regions were identified as hotspots for oxidative damage, with lower levels of oxidized mtDNA observed in the enalapril group (*p* = 0.0146 and 0.0354 for Cytb and D-Loop, respectively; Figure 4).

### 3.3. Protein Expression of Selected Mediators of Energy Metabolism, Mitochondrial Biogenesis, Dynamics and Autophagy

**Figure 5:**
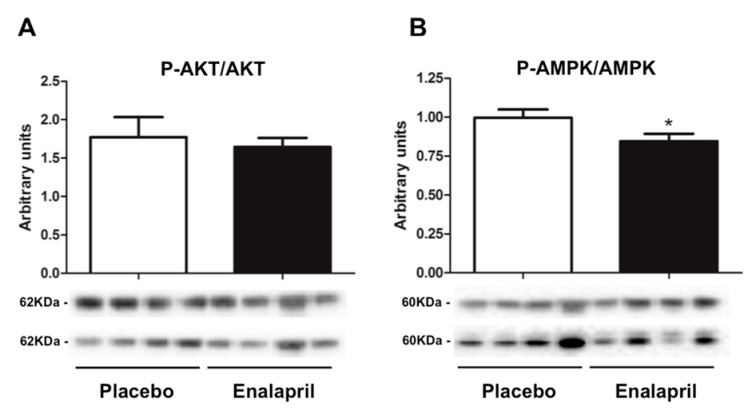
Protein expression of the active form of (**A**) protein kinase B (AKT) and (**B**) AMP-activated protein kinase (AMPK) in heart samples of old rats treated with placebo (*n* = 8) or enalapril (*n* = 8). Data are shown as the ratio between the phosphorylated (upper bands) and non-phosphorylated (lower bands) form of the two proteins. Bars represent mean values (± standard error of the mean) in the two experimental groups. Values are expressed in arbitrary units. Representative blots are shown. * *p* = 0.0408 vs. placebo.

We started our analysis with the evaluation of mediators involved in cellular energy metabolism. To this aim, we analyzed the expression of the conserved fuel-sensing enzyme AMPK and its upstream activator, serine-threonine protein kinase AKT. A trend toward decreased content of both mediators in the hearts of enalapril-treated rats was observed (Figure 5); however, only AMPK reached the statistical significance (*p* = 0.0408).

**Figure 6:**
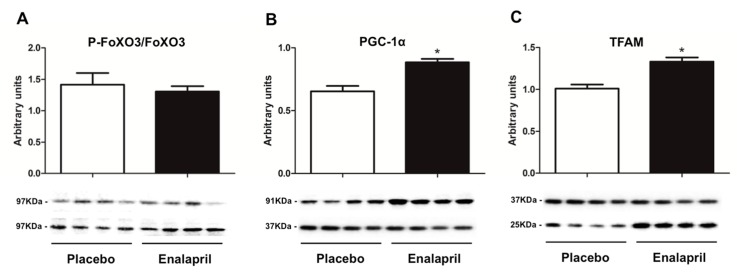
Protein expression of (**A**) Forkhead boX O3 (FoXO3), (**B**) peroxisome proliferator-activated receptor gamma coactivator 1-alpha (PGC-1α), and (**C**) mitochondrial transcription factor A (TFAM) in heart samples of old rats treated with placebo (*n* = 8) or enalapril (*n* = 8). FoXO3 expression is shown as the ratio between the phosphorylated (upper bands) and non-phosphorylated form (lower bands). Bars represent mean values (± standard error of the mean) relative to the placebo group. Values are expressed in arbitrary units. Representative blots are shown. * *p* < 0.0001 vs. placebo.

**Figure 7:**
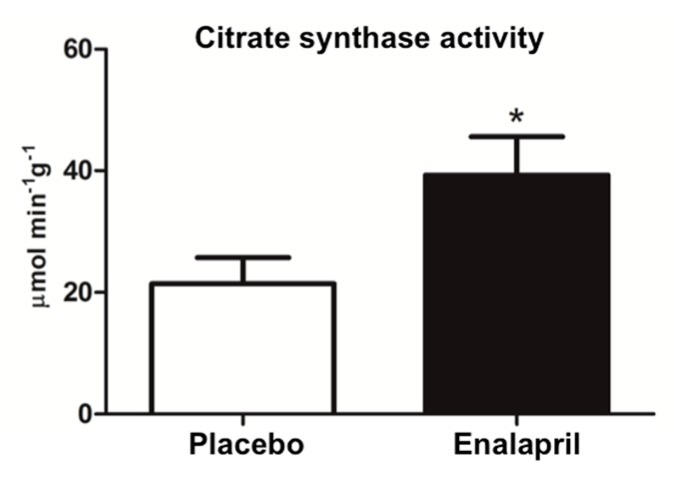
Citrate synthase activity in heart samples of old rats treated with placebo (*n* = 8) or enalapril (*n* = 8). Bars represent mean values (± standard error of the mean) in the two experimental groups. Values are expressed in µmol × min^−1^ × g tissue^−1^. * *p* = 0.0258 vs. placebo.

Mitochondrial biogenesis signaling was then analyzed via the determination of the protein expression of selected mediators including FoXO3 and its ser253 phosphorylated form (FoXO3-P), PGC-1α, and TFAM. CS activity was also measured as a marker of mitochondrial mass. FoXO3 expression was unvaried between groups (*p* = 0.6043; Figure 6A). Instead, the content of PGC-1α and TFAM was greater in rats receiving enalapril compared with controls (*p* < 0.0001; Figure 6B,C), suggesting upregulation of mitochondrial biogenesis under ACE inhibition. Accordingly, CS activity was higher in enalapril-treated rats relative to the placebo group (*p* = 0.0258; Figure 7).

**Figure 8:**
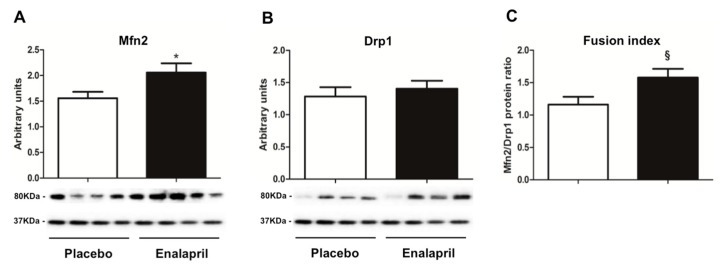
Protein expression of (**A**) mitofusin 2 (Mfn2) and (**B**) dynamin-related protein (Drp1), and (**C**) the fusion index (Mfn2/Drp1) in heart samples of old rats treated with placebo (*n* = 8) or enalapril (*n* = 8). Bars represent mean values (± standard error of the mean) in the two experimental groups. Values are expressed in arbitrary units. Representative blots are shown. * *p* = 0.0335 vs. placebo; ^§^ *p* = 0.0320 vs. placebo.

The protein expression of Mfn2 and Drp1 was measured to obtain indications on mitochondrial dynamics signaling in the two experimental groups. The protein content of Mfn2 was higher in the enalapril-treated rats (*p* = 0.0335; Figure 8A), whilst Drp1 expression did not vary between groups (*p* = 0.5237; Figure 8B). As a consequence, the fusion index, calculated as the ratio between Mfn2 and Drp1, was higher in the heart of old rats treated with enalapril (*p* = 0.0320; Figure 8C). This finding suggests a shift of mitochondrial dynamics signaling toward fusion following enalapril administration.

**Figure 9:**
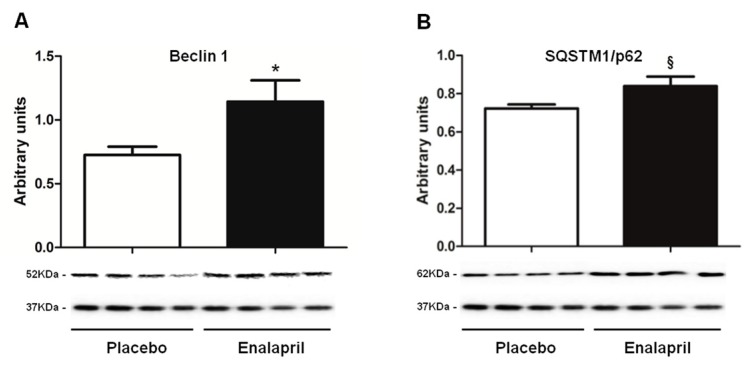
Protein expression of Beclin 1 and sequestome 1 (SQSTM1)/p62 in heart samples of old rats treated with placebo (*n* = 8) or enalapril (*n* = 8). Bars represent mean values (± standard error of the mean) in the two experimental groups. Values are expressed in arbitrary units. Representative blots are shown. * *p* = 0.0471 *p* = 0.0346 vs. placebo.

Finally, we evaluated the expression of two mediators of autophagy: Beclin 1 and SQSTM1/p62. The former is the mammalian ortholog of Atg6/vacuolar protein sorting 30 in yeast. Beclin 1 is involved in autophagosome formation, cargo recruitment and autophagosome maturation. SQSTM1/p62, a ubiquitin-binding scaffold, is a receptor of autophagy that colocalizes with ubiquitinated protein aggregates. Higher protein levels of both Beclin 1 and SQSTM1/p62 were found in the enalapril group compared with controls (*p* = 0.0471 and *p* = 0.0346, respectively; Figure 9), suggesting that ACE inhibition might impact the autophagic process.

### 3.4. Analysis of Mitochondrial Transcription Factor A Binding to Specific Regions of Mitochondrial DNA

Based on previous findings reporting an age-related modulation of TFAM binding to mtDNA in several rat tissues [31], we carried out mIP experiments and analyzed the binding of TFAM to three functionally relevant mtDNA regions by qRT-PCR. Such regions encompassed: (1) the portion of the D-loop including the light strand transcription promoter (LSP); (2) the tRNA genes stretch enclosing OriL; and (3) a sequence containing the direct repeat 1 (DR1) of the 4.8-kb deletion.

The D-loop- and the OriL-containing regions were selected because of their role in mtDNA replication, involving TFAM binding. The region including DR1 was chosen because of the possible involvement of TFAM in the generation of mtDNA deletions [33,34]. The tested sequences were far enough from each other (600 bp on average) to rule out the possibility of false positive results deriving from the amplification of overlapping sequences physically immunoprecipitated by the same TFAM molecule [35].

**Figure 10:**
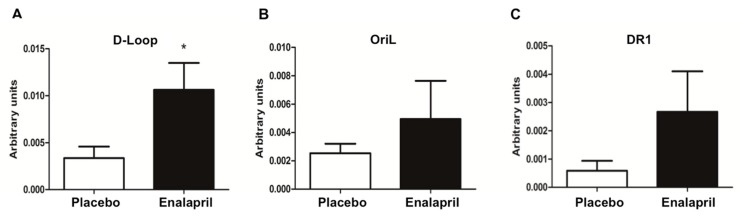
Determination of TFAM binding to mtDNA regions encompassing D-Loop (**A**), OriL (**B**) and DR1 (**C**) in heart samples of old rats treated with placebo (*n* = 8) or enalapril (*n* = 8). Bars represent mean values (± standard error of the mean) in the two experimental groups. Values are expressed in arbitrary units. * *p* < 0.0452 vs. placebo.

The mean amount of TFAM-bound mtDNA in the D-loop region was more than three-fold greater in enalapril-treated rats relative to those receiving placebo (*p* = 0.0452; Figure 10A). A similar trend was observed for the OriL and DR1 regions (two-fold and four-fold, respectively) (Figure 10B,C). However, because of the large within-group variability, the differences did not reach the statistical significance (*p* = 0.4269 and 0.217 for OriL and DR1, respectively; Figure 10B,C).

## 4. Discussion

The present study was designed to explore the effects of non-antihypertensive enalapril administration started late in life on mitochondrial genomic stability, TFAM binding to mtDNA, and MQC signaling in the heart of old rats. We started our experimental plan by measuring mtDNA content. To gather insights into the impact of enalapril treatment on mitochondrial mass and mtDNA homeostasis, we measured CS activity, the abundance of oxidative lesions and mtDNA^4834^ deletion, and the binding of TFAM to specific regions of mtDNA involved in transcription and deletion generation. Finally, we explored the effect of enalapril administration on MQC signaling by estimating the expression of antioxidant enzymes and selected mediators of mitochondrial biogenesis, dynamics, and autophagy.

Rats treated with enalapril showed a reduced heart weight either absolute or adjusted by BW (Table 3), indicative of mitigation of age-related cardiac hypertrophy. A previous study conducted by our group using a similar experimental model showed that enalapril administration did not affect locomotor activity at any time during the three months of treatment relative to placebo [20]. This rules out the possibility that the reduction of the heart weight in enalapril-treated rats may be attributed to decreased activity levels. Higher mtDNA copy number was found in the heart of rats treated with enalapril (Figure 3A). Even though this adaptation could seem beneficial, it might be detrimental if the proportion of damaged molecules is not reduced. Indeed, deleted mtDNA can accumulate by virtue of a replicative advantage over wild type molecules [36]. In our experimental model, the administration of enalapril was associated with a lower abundance of mtDNA^4834^ deletion (Figure 3B) and a reduced severity of oxidative lesions to functional regions of mtDNA (Cytb and D-Loop) (Figure 4). Collectively, these findings support a protective effect of enalapril with regard to mitochondrial genome stability in the aged heart.

As a further indication of increased mitochondrial mass under ACE inhibition, we determined higher CS activity in enalapril-treated rats (Figure 7). This adaptation was likely sustained by stimulation of mitochondriogenesis via upregulation of PGC-1α and TFAM (Figure 6B,C) [37]. PGC-1α is a transcriptional coactivator that stimulates mitochondrial proliferation in states of increased energy demand. The latter is sensed as a higher AMP/ATP ratio by AMPK, an upstream regulator of PGC-1α that is activated by phosphorylation [38]. However, different from PGC-1α, AMPK activation was blunted by enalapril (Figure 5B). A similar pattern was also determined for AKT (Figure 5A). These counterintuitive findings might be explained by the reduced oxidative burden detected in the heart of enalapril-treated rats (Figure 2 and Figure 4). Indeed, the activation of both AMPK and AKT is promoted by oxidative stress [39,40]. This hypothesis is in line with our previous observation of lower hydrogen peroxide generation by subsarcolemmal mitochondria in skeletal muscle of old rats treated with enalapril [21]. The upregulation of the expression of the mitochondrial detoxifying enzymes PrxIII and MnSOD (Figure 1A,C) lends further support to the cardioprotective effects of enalapril via mitigation of oxidative stress. Notably, the expression of PrxIII and MnSOD in the heart is induced by PGC-1α [41], therefore establishing a virtuous circle involving stimulation of mitochondrial biogenesis and ROS scavenging systems.

The signaling cascade of PGC-1α proceeds through the activation of nuclear respiratory factors that promote the expression of a set of nuclear-encoded mitochondrial proteins [37]. Among these, TFAM, a histone-like protein involved in mtDNA replication and transcription, is thought to regulate mitochondriogenesis via binding to mtDNA [37]. This hypothesis is supported by the finding of increased binding of TFAM to regions that control mtDNA replication (Figure 10A–C). Interestingly, in the same regions, levels of oxidized mtDNA were lower in enalapril-treated rats compared with controls (Figure 4). Since TFAM binds more avidly to oxidized bases, thereby possibly hindering mtDNA repair and replication [19], the reduction in mtDNA oxidation promoted by enalapril provides an additional explanation to the upregulation of mitochondrial biogenesis under ACE inhibition.

Further adaptations induced by enalapril involved mitochondrial dynamics signaling. Indeed, enalapril-treated rats showed increased protein expression of the fusion protein Mfn2 (Figure 8A), whereas Drp1 protein content was unaffected by the treatment (Figure 8B). The consequent increase in the fusion index (Figure 8C) suggests that enalapril might promote a shift of mitochondrial dynamics towards fusion. It has been hypothesized that this adaptation could serve as a mechanism whereby the functionality of damaged mitochondria can be complemented and possibly restored by their fusion with neighboring intact organelles [42]. This might be achieved through the dilution of mtDNA damage along the network [43]. In support to this idea, disruption of mitochondrial fusion via Mfn ablation was found to increase mitochondrial dysfunction and lethality in mtDNA-mutator mice [44]. Furthermore, while inhibiting mitochondrial fusion impairs myocardial contractility [45,46,47], the inhibition of mitochondrial fission is cardioprotective against ischemia/reperfusion injury in murine models [48]. This is achieved through the formation of elongated mitochondria under AKT signaling [49].

However, sustained upregulation of mitochondrial fusion may also have drawbacks such as inhibition of autophagy. Indeed, damaged mitochondria within the network may be hard to single out by fission, thereby hindering their autophagic disposal [50]. In this scenario, while the increased expression of Beclin 1 in rats treated with enalapril (Figure 9A) might suggest an induction of autophagy, the greater SQSTM1/p62 content found in the same rat group (Figure 9B) may be indicate of defective autolysosome clearance. However, our results offer an alternative explanation for which upregulation of Beclin 1 and SQSTM1/p62 might reflect greater ongoing autophagy at the time of tissue collection. The actual meaning of the pattern of expression of the two autophagy mediators cannot be discerned through our experimental design and warrants further investigation.

Albeit reporting novel findings, our study presents some limitations that deserve being acknowledged. All of our biochemical assessments were conducted at a single time-point. Hence, no information on the time course of the pathways under investigation could be provided. The amount of cardiac tissue available was insufficient for measuring the expression and activity of other relevant mediators of MQC (e.g., microtubule-associated protein 1 light chain 3 beta (LC3B)), ROS production, and mtDNA repair enzymes. This, together with the lack of tissue imaging analysis investigating the extent of tissue fibrosis and cardiac functional assessment, does not allow for drawing definitive conclusions on the nature and extent of adaptations elicited by enalapril. However, our experimental plan allowed for interrogating a fairly comprehensive range of biochemical events and signaling pathways relevant to cardiac aging. The present data may therefore pave the way for future studies aimed at dissecting individual adaptations elicited by ACE-inhibitors that might be targeted to achieve better cardiac health in advanced age.

## 5. Conclusions

Findings from the present study indicate that late-life enalapril administration at a non-antihypertensive dose attenuates age-associated cardiac hypertrophy and oxidative stress-related molecular damage (i.e., mtDNA oxidation, mtDNA^4834^ deletion, and protein carbonylation), while upregulating mitochondrial enzymatic antioxidant defenses. The binding of mitochondrial transcription factor A to mtDNA regions involved in replication and deletion generation was greater in rats treated with enalapril. The same rat group showed increased indices of mitochondrial mass, upregulation of mitochondrial biogenesis signaling, and enhanced mitochondrial fusion signaling. Finally, treatment with enalapril was associated with an increased protein content of Beclin 1 and SQSTM1/p62.

Given the wide range of adaptations elicited by enalapril and their potential relevance to cardioprotection, further research is warranted to finely dissect the biochemical pathways modulated by ACE inhibitors and to establish if these drugs may be used to preserve cardiac health into late life.

## Figures and Tables

**Table 1:** Technical specifications of the primary antibodies used for Western immunoblotting.

Antibody	Manufacturer and Catalogue Number	Type	Species	Dilution	Detected Band MW (kDa)
Anti AKT	Cell Signaling (Berverly, MA, USA)	Polyclonal	Rabbit	1:20,000	60
	(sc-9272)				
Anti AMPK	Cell Signaling	Polyclonal	Rabbit	1:5000	62
	(sc-2532)				
Anti Beclin 1	Millipore (Burlington, MA, USA)	Polyclonal	Rabbit	1:300,000	52
	(AB15417)				
Anti Drp1	Abnova (Taiwan, China)	Monoclonal	Mouse	1:10,000	80
	(H00010059-M01)				
Anti FoxO3a	Cell Signaling	Polyclonal	Rabbit	1:10,000	97
	(sc-9467)				
Anti MFN2	Abnova (Taiwan, China)	Monoclonal	Mouse	1:10,000	80
	(H00009927-M03)				
Anti MnSOD	Assay Designs (Farmingdale, NY, USA)	Polyclonal	Rabbit	1:40,000	26
	(SOD-110)				
Anti P-AKT	Cell Signaling	Polyclonal	Rabbit	1:5000	60
	(sc-9271)				
Anti P-AMPK	Cell Signaling	Polyclonal	Rabbit	1:5000	62
	(sc-2531)				
Anti P-FoxO3a	Cell Signaling	Polyclonal	Rabbit	1:5000	97
	(sc-9465)				
Anti PGC-1α	Santa Cruz Biotechnology (Dallas, TX, USA)	Polyclonal	Goat	1:10,000	91
	(sc-5816)				
Anti PrxIII	Ab Frontier (Seoul, South Korea)	Polyclonal	Rabbit	1:160,000	26
	(LF-PA0030)				
Anti Prx-SO_3_	Ab Frontier (Seoul, South Korea)	Polyclonal	Rabbit	1:30,000	26
	(LF-PA0004)				
Anti SQSTM1/p62	Sigma-Aldrich (St. Louis, MO, USA)	Monoclonal	Rabbit	1:20,000	62
	(P0066)				
Anti TFAM	Santa Cruz Biotechnology (Dallas, TX, USA)	Polyclonal	Goat	1:30,000	25
	(sc-19050)				
Anti GAPDH	Cell Signaling	Monoclonal	Rabbit	1:100,000	37
	(sc-2118)				

AKT, protein kinase B; AMPK, AMP-activated protein kinase; Drp1, dynamin-related protein 1; FoXO3a, Forkhead boX O3a; GADPH, glyceraldehyde 3-phosphate dehydrogenase; Mfn2, mitofusin 2; MnSOD, manganese superoxide dismutase; MW, molecular weight; P-AKT, phosphorylated AKT; P-AMPK, phospho-AMPK; P-FoXO3a, phosphorylated FoXO3a; PGC-1α, eroxisome proliferator-activated receptor gamma coactivator 1-alpha; PrxIII, peroxiredoxin III; Prx-SO_3_, oxidized PrxIII; SQSTM1/p62, sequestosome 1 p62; TFAM, mitochondrial transcription factor A.

**Table 2:** Oligonucleotide primer sequences.

Primer Set	Forward Primer	Reverse Primer	(nps)	(nps)
β-actin	5′CCCAGCCATGTACGTAGCCA3′	5′CGTCTCCGGAGTCCATCAC3′	2181–2200	2266–2248
D-loop	5′CACCCCCTACACCTGAAACTT3′	5′TTTGTGTCGGGAAATTTTACCAAT3′	16,092–16,112	16,250–16,227
mtDNA	5′GGTTCTTACTTCAGGGCCATCA3′	5′TGATTAGACCCGTTACCATCGA3′	15,785–15,806	15,868–15,847
mtDNA^4834^	5′AAGGACGAACCTGAGCCCTAATA3′	5′CGAAGTAGATGATGCGTATACTGTA3′	8109–8131	13,020–12,996
ND1	5′AACGCCCTAACATCAATTGTATTCC3′	5′TGGTCATATCGAAAACGGGGG3′	3442–3466	3590–3570
OriL	5′CAGCTAAATACCCTACTTACTGG3′	5′GCCCCCTTTTTACCAAAAAGCC3′	5120–5142	5270–5249

Nucleotide numbering is reported according to the GenBank accession number AY172581 (*Rattus norvegicus*, complete mitochondrial genome), except for the β-actin primer set which is reported according to GenBank accession number V01217.1 (*Rattus norvegicus*, β-actin gene). nps: nucleotide positions, mtDNA: mitochondrial DNA.

**Table 3:** Morphological characteristics of experimental animals at the time of sacrifice.

	Placebo (*n* = 8)	Enalapril (*n* = 8)	*p*-Value
Body weight, g	577.3 ± 12.9	551.0 ± 16.3	0.2228
Heart weight (absolute), g	1.53 ± 0.03	1.37 ± 0.05	0.0359
Heart weight by body weight	2.92 ± 0.06	2.63 ± 0.10	0.0359

Data are shown as mean ± standard error of the mean.
